# Concomitant Amyotrophic Lateral Sclerosis and Rheumatoid Arthritis: A Case Report

**DOI:** 10.7759/cureus.74301

**Published:** 2024-11-23

**Authors:** Mustafa Alhayali

**Affiliations:** 1 Internal Medicine/Rheumatology, Ibn Sina University for Medical and Pharmaceutical Sciences, Baghdad, IRQ; 2 Rheumatology and Medical Rehabilitation, Center of Spine and Joint Diseases, Baghdad, IRQ

**Keywords:** amyotrophic lateral sclerosis, methotrexate, motor neuron disease, rare association, rheumatoid arthritis

## Abstract

Amyotrophic lateral sclerosis (ALS) is a progressive neurodegenerative motor neuron disease that leads to a gradual loss of motor neurons manifesting as progressive weakness, dysarthria, and respiratory decline, with a relatively short life expectancy. Rheumatoid arthritis (RA) is an autoimmune disorder characterized by polyarthritis and affects multiple systems. Motor neuron involvement is rare in rheumatoid arthritis. Here, we report a unique case of a patient with an established diagnosis of ALS who later developed seropositive RA. A 58-year-old male from Baghdad presented to our center with polyarticular joint pain, stiffness, and swelling for about four months, the patient had a history of progressive neurological deficits. The final diagnosis was seropositive rheumatoid arthritis with concomitant amyotrophic lateral sclerosis. While the patient's joint symptoms responded well to methotrexate and prednisolone, he continued to experience a neurological decline. This is one of the few reported cases of concurrent ALS and RA, highlighting the complexity of managing overlapping neurodegenerative and autoimmune conditions.

## Introduction

Amyotrophic lateral sclerosis (ALS) is a progressive neurodegenerative motor neuron disease characterized by loss of motor neuron cells leading to gradual limb weakness, dysarthria, dysphagia, and eventually respiratory failure with a high mortality rate [1]. The incidence of ALS ranges between 1.5 and 2.5 for 100,000 per year. Although familial cases of ALS exist, about 90% are sporadic with an unclear etiology [2].

Rheumatoid arthritis (RA) is an autoimmune disorder affecting multiple systems, primarily characterized by polyarthritis [3]. Neurologic complications are reported in about 40% of RA patients and can include various types of peripheral neuropathy, such as mononeuritis multiplex or sensory and motor polyneuropathy, cervical myelopathy due to spinal cord compression, entrapment neuropathy, such as carpal tunnel syndrome, and central nervous system involvement primarily due to vasculitis [4]. Motor neuron involvement, however, is rare in RA.

To the best of our knowledge, there are only a few described cases of concomitant ALS and RA, one of which occurred during anti-tumor necrosis factor inhibitor therapy [5]. Here, we report a patient with a previous diagnosis of ALS who subsequently developed seropositive RA, a unique presentation that offers insights into the complex interactions of neurodegenerative and autoimmune diseases.

## Case presentation

A 58-year-old male from Baghdad presented to our center with progressive joint pain involving both hands, elbows, and feet that had persisted for approximately four months. He reported experiencing significant morning stiffness lasting around two hours along with gradual swelling of both hands. Systemic review revealed the presence of progressive exertional dyspnea and chronic constipation. The patient had a 14-year history of diabetes mellitus, he also had a history of progressive upper and lower limb weakness and heaviness for the last 12 months leading to a diagnosis of ALS.

**Figure 1:**
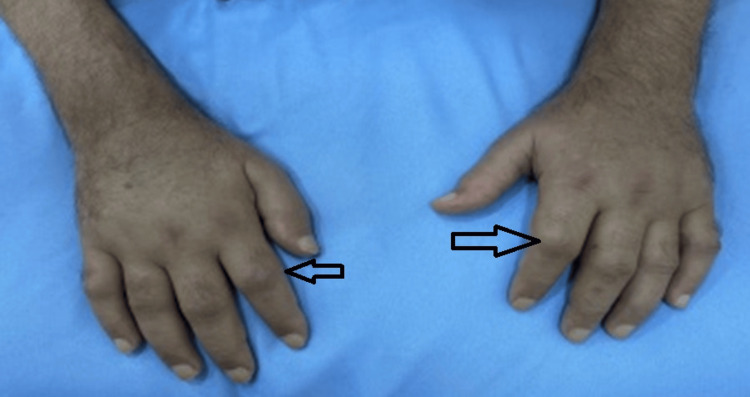
Swelling of small joints of both hands (arrows)

On physical examination, notable swelling was observed in the wrists, metacarpophalangeal joints, and proximal interphalangeal joints (Figure 1). Additionally, tenderness was present over the metatarsal bones, as well as in the knees and elbows.

Neurological examination revealed diffuse muscle wasting, fasciculations over both thighs triggered by flickering, hypertonia of upper and lower limbs, proximal and distal muscle weakness, hyperreflexia of knee and ankle jerks with normal upper limbs reflexes, and slow rapid alternating movements. Sensory and cranial nerve examination was not significant. The rest of the physical examination was not significant. Initial laboratory tests are shown in Table 1.

**Table 1:** Baseline investigations at the initial visit

Test	Result	Reference Range
White blood cells (WBC)	7.7x10^9^	4-10x10^9^
Hemoglobin (Hb)	9.8 g/dl	12-16g/dl
Platelets	559x10^9^/L	150-400x10^9^/L
Erythrocyte sedimentation rate	98mm/hour	0-30mm/hour
C-reactive protein	67.5mg/L	<6 mg/L
Rheumatoid factor	164 U/ml	<20 U/ml
Anti-citrullinated peptide antibody	202 U/ml	<30 U/ml
Alanine aminotransferase	59 IU/L	<40 IU/L
Aspartate aminotransferase	29 IU/L	<33 IU/L
Creatinine	0.77 mg/dL	0.7-1.4 mg/dL

**Figure 2:**
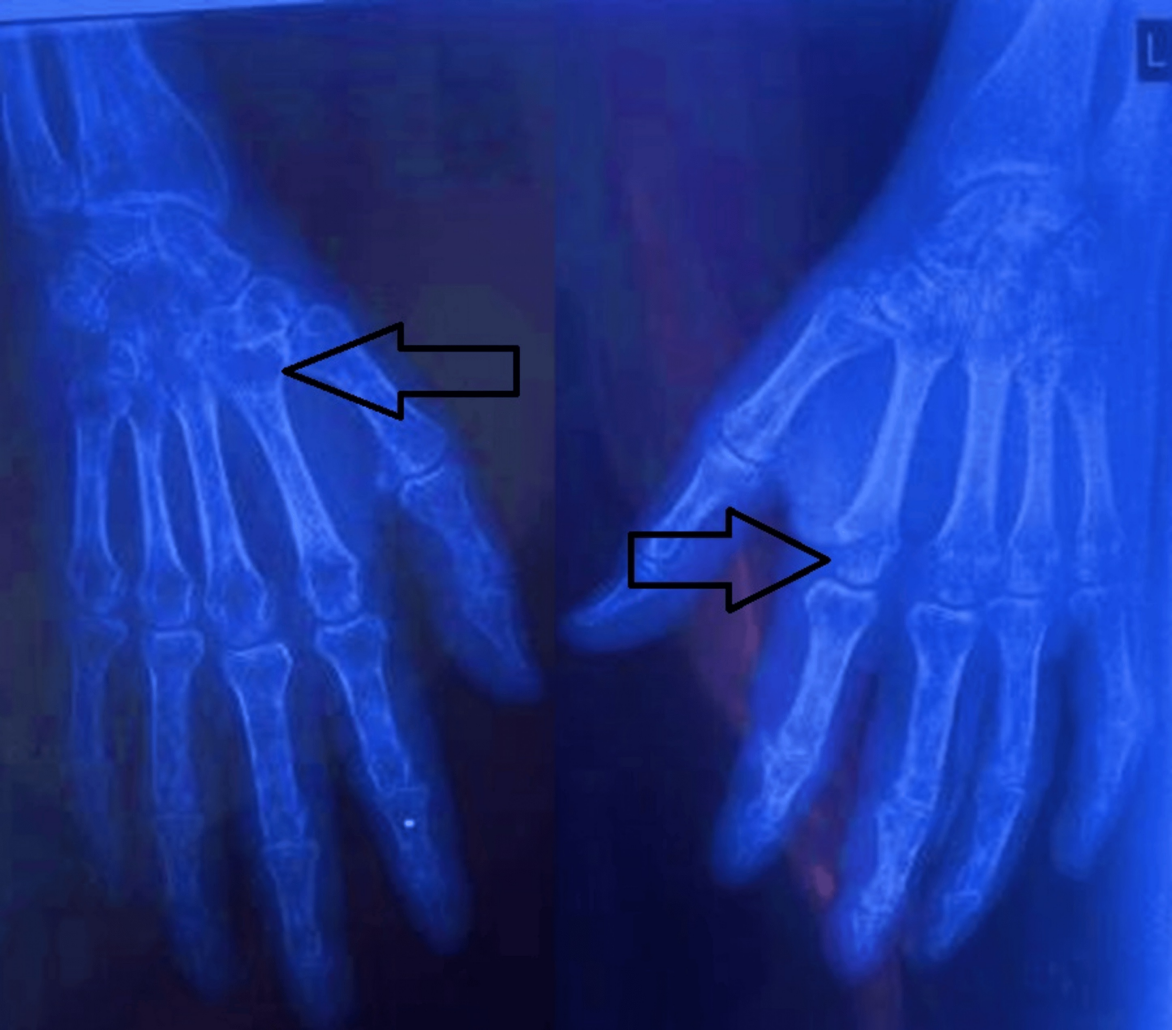
Hands radiographs showing periarticular osteopenia and erosions (arrows)

Hand radiographs showed periarticular osteopenia and erosive changes in the wrists and small hand joints (Figure 2).

The patient was on a regimen of metformin 500 mg twice daily for diabetes, riluzole 50 mg twice daily for ALS, and a daily multivitamin. Based on the 2010 American College of Rheumatology (ACR)-European Alliance of Associations for Rheumatology (EULAR) classification criteria for RA [6], the patient was diagnosed with seropositive rheumatoid arthritis, with a Clinical Disease Activity Index (CDAI) of 18 indicating moderate disease activity. Initial treatment included intramuscular methotrexate 20 mg weekly, folic acid 5 mg weekly, prednisolone 10 mg daily, and etoricoxib 60 mg daily. After one month of treatment, the patient reported significant improvement in joint symptoms, and laboratory results showed a notable reduction in inflammatory markers (Table 2), consequently, corticosteroid tapering was initiated. Follow-up visits confirmed a sustained improvement in RA symptoms, with the CDAI score reflecting continued disease control. However, there was a progressive decline in muscle strength accompanied by worsening dyspnea, underscoring the ongoing impact of ALS on the patient's condition.

**Table 2:** Laboratory findings after treatment

Test	Result	Reference Range
White blood cells (WBC)	6.1x10^9^	4-10x10^9^
Hemoglobin (Hb)	9.9 g/dl	12-16g/dl
Platelets	312x10^9^/L	150-400x10^9^/L
Erythrocyte sedimentation rate	38 mm/hour	0-30mm/hour
C-reactive protein	6.4mg/L	<6 mg/L
Alanine aminotransferase	47 IU/L	<40 IU/L
Aspartate aminotransferase	28 IU/L	<33 IU/L
Creatinine	0.9 mg/dL	0.7-1.4 mg/dL

## Discussion

There are only a few case reports of patients with an initial diagnosis of rheumatoid arthritis who were then diagnosed with ALS. To the best of our knowledge, this is the first published case of ALS, who later on developed seropositive rheumatoid arthritis.

ALS is a chronic, incurable, life-threatening disease with a median survival rate of three to five years [7]. Clinically, the presence of both upper and lower motor neuron symptoms and signs is the hallmark of the disease, these features may produce impairments affecting limbs, bulbar, axial, and/or respiratory function [8]. The pathogenesis of neurological involvement in different rheumatic conditions is still unclear; however, studies to explain this have mainly concentrated on the effect of autoantibodies, pro-inflammatory cytokines, chemokines, and other causes of blood-brain barrier defects [9]. It is thought that inflammation in the blood-brain barrier eases the transport of autoantibodies to the cerebrospinal fluid; these changes result in central nervous system involvement in some rheumatic conditions. Moreover, vasculitis has a significant role in affecting the nervous system [10-12].

In addition, cytokines and chemokines enhance the effect of Th1 and B cells, thereby increasing the neurologic involvement in some autoimmune disorders [13]. C4 levels in the sera of patients with ALS were lower than normal in a study conducted by Wang et al. [14]. This may cause immune dysregulation and raise the risk of developing other autoimmune diseases. Based on these immunological changes in ALS, some studies are being conducted to assess the role of immune-modulating therapies in the treatment of ALS [15,16].

Although the patient's neurological diagnosis preceded the clinical presentation of rheumatoid arthritis, it is possible that subclinical inflammation or “preclinical RA” was present for a period and caused blood brain barrier defect. The concurrent appearance of the two conditions makes the diagnosis challenging because RA may resemble some musculoskeletal manifestations of ALS. The short life span of patients with ALS makes it harder to determine the relationship between these two diseases. There is no evidence for a common pathophysiologic mechanism, and thus the possibility of a co-incident association may be raised. Due to the neurological side effects of some anti-rheumatoid drugs, the treatment of concurrent RA and ALS represents another challenge. Among these treatments, anti-tumor necrosis factor alpha (TNFa) is commonly used in the treatment of rheumatoid arthritis and other forms of arthritis. Among the serious adverse effects, neurologic diseases, including demyelination and multiple sclerosis (MS)-like syndromes, have been reported [17]. Moreover, anti-TNFa therapy causes worsening in MS patients indicating a causative relationship [18]. Fortunately, our patient responded well to methotrexate monotherapy eliminating the need for more aggressive treatment.

A case of overlap between rheumatoid arthritis, SLE, and Sjogren’s syndrome complicated by motor neuron disease was reported by Ebru Atalar et al. [19]. Melissa Padovan reported two cases of RA patients developing ALS years after the diagnosis of RA [20]. Another reported case of concomitant RA and ALS has been reported by M’Bappe et al. [21]. In contrast to these cases, our patient developed RA after being diagnosed with ALS.

## Conclusions

This case highlights the possible correlation between RA and ALS. The presence of these entities in the same patient may be co-incidental or there is an actual correlation between them. Neurological manifestations can be found in rheumatoid patients, but the presence of fasciculations and mixed upper and lower motor neuron signs should suggest amyotrophic lateral sclerosis. It is known that some patients with rheumatic conditions first present at neurology clinics with neurological symptoms and vice versa. It is crucial to monitor and investigate patients with rheumatoid arthritis for neurological manifestations and patients with ALS for joint symptoms.
